# Research on Carbon Emissions Reduction Strategy Considering Government Subsidy and Free Riding Behavior

**DOI:** 10.1089/ees.2021.0192

**Published:** 2022-04-13

**Authors:** Haixia Gui, Jing Xue, Yun Li, Liangcheng Chen

**Affiliations:** School of Economics and Management, Anhui University of Science and Technology, Huainan, China.

**Keywords:** carbon emissions reduction, evolutionary game, free riding behavior, government subsidy

## Abstract

Government subsidy can greatly encourage supply chain enterprises to reduce carbon emissions. To quickly occupy the market, supply chain enterprises form alliances. However, enterprises in the alliance have speculative psychology, and the impact of such free riding behavior on the carbon emissions reduction willingness of supply chain enterprises is still unclear. In this article, government subsidies and free riding behavior parameters are introduced to build a carbon emissions reduction decision model for the government, manufacturers, and suppliers, and the impact of government subsidies and free riding behavior on the decision making of supply chain enterprises is analyzed through evolutionary game theory. The analysis shows that government subsidies have an incentive effect on carbon emissions reduction of supply chain enterprises. After the market stabilizes, even if the government subsidies are gradually withdrawn, the carbon emissions reduction of supply chain enterprises still converges to Pareto optimal equilibrium. The influence of free riding behavior on supply chain enterprises depends on the carbon emissions reduction profit. When the carbon emissions reduction profit is different, the decision of manufacturers and suppliers will be different. The above conclusions provide a reference for governments to strengthen control or enterprises to make decisions on carbon emissions reduction.

## Introduction

In recent years, low-carbon development, energy conservation, and emissions reduction have become a common understanding of all countries due to global warming. The increase of carbon emissions is mainly attributed to unreasonable energy consumption and broad economic growth (Chen *et al.,* 2016). The central economic conference in December 2020 identified carbon peak and carbon emissions as one of the eight key tasks of the CPC Central Committee in 2021 (Zhou and Wang*,* 2020). To promote the development of low-carbon economy, the government has issued various environmental laws and regulations and preferential policies to enhance the enthusiasm of enterprises to reduce carbon emissions. The implementation of environmental protection measures and the consumers' preference for low-carbon products incent manufacturers and suppliers to form a supply chain alliance (Liu *et al.,* 2021).

Matthews *et al.* (2009) pointed out that when carbon emissions reduction is carried out, it is difficult to achieve it through the efforts of a single entity, and only the coordinated management of upstream and downstream enterprises in the supply chain can be promoted (Ji *et al.,* 2017). For example, after Walmart (He, 2014) joined the carbon disclosure program in 2005, it was found that 90% of the carbon emissions in its supply chain came from its suppliers. Walmart provided assistance in production process and technical guidance on carbon emissions reduction to its cotton thread suppliers by making use of its strong network, so as to reduce the carbon emissions of the enterprises. As the policy guidance and encouraging measures are one of the main factors for the supply chain enterprises to reduce carbon emissions, the game between the government and the supply chain enterprises has an important impact on the decision making by the supply chain enterprises.

However, they ignore the fact that in a supply chain alliance, a certain unit will be speculative (Tian *et al.,* 2018), ship by dint of strength, enjoy the benefit of the group without bearing the cost (Jing and Meng*,* 2019), and excessive free riding will reduce the incentive for enterprises to reduce carbon emissions (Gong *et al.,* 2020). However, Zheng *et al.*(2020) and Ashkan (2018) found that government subsidy makes supply chain enterprises reach equilibrium faster, which can promote supply chain enterprises to carry out carbon emissions reduction to some extent, and reduce the impact of free riding behavior (Ping *et al.,* 2020).

Based on this, this article constructs a tripartite game model of government, manufacturers, and suppliers, considers the free riding behavior of manufacturers and suppliers, studies the decision making of carbon emissions reduction of supply chain enterprises with the participation of government, and determines the evolutionary game strategy of their carbon emissions reduction behavior, the sensitivity of evolutionary stable game strategy, and free riding behavior parameters is analyzed numerically.

## Literature Review

To better explain the motivation and research background of this study, the relevant literature is reviewed from two aspects of the influence of free riding behavior and government subsidy on supply chain enterprises' decision making.

Telser (1960) first proposed that there is free riding behavior in the alliance, and pointed out that free riding behavior would affect the decision making of the enterprise. Scholars build different types of game models to verify the influence of free riding behavior on participants' decisions. For example, Guo *et al.* (2021) took the apparel supply chain as an example, constructing a tripartite game model among the government, manufacturers, and suppliers, and concluding that free riding behavior would affect the carbon emissions reduction decisions of enterprises. Pu *et al.* (2017) constructed an online/offline dual-channel model and found that the level of sales effort and supply chain profit decrease with the increase of free riding. Yuan *et al.* (2018) built a game model of cooperation between manufacturers and suppliers and found that the free riding effect would affect the decision making by the supply chain enterprises, but the expected revenue was greater compared with the decentralized enterprises.

For a deeper understanding of how free riding behavior affects participants' decisions, Liu *et al.* (2020) compared the profit model with or without free riding and found that free riding increases the total market demand by influencing pricing, but produced segmentation effects on offline demand. Ke and Jiang (2020) model supply chain marketing strategies of retailers with different strengths and found that for dominant firms, if free riding was not serious, efforts would be made. The strategy will vary according to the degree of free riding in the dominant structure, usually in a supply chain alliance, manufacturers dominated (Zhou *et al.,* 2018). For the suppliers, the only time they are willing to cut carbon emissions is when the profit of doing so is above a certain threshold (Li *et al.,* 2016).

Although there are many researches on free riding behavior, most of them focus on the influence of free riding behavior on the decision making of supply chain enterprises in a completely free market, ignoring the regulatory role of the government.

In the market economy, the government has a great influence on carbon emissions reduction, which can effectively promote the carbon emissions reduction of enterprises to a certain extent (Kushwaha *et al.,* 2020; Yao *et al.,* 2020). It can be seen that carbon emissions reduction not only involves the price of endogenous variables, but also relates to the regulatory role of the government in the market. Scholars discuss the impact of government participation on corporate carbon emissions reduction decision making in different ways of government participation. Xuan *et al.* (2020) collected the panel data of 30 Chinese provinces from 2000 to 2016 and used DID method to explore whether government participation has a significant promoting effect on carbon emissions reduction. Xie *et al.* (2020) introduced carbon quota and carbon emissions trading policies into the dual-channel supply chain model and believed that the government designed incentive measures to regulate the carbon market. Yu *et al.* (2020) analyzed the game relationship between the government, enterprises, and investors from the perspective of regulation, and believed that governments should engage in low-carbon markets and regulate them through policies. Zhu *et al.* (2018) and He *et al.* (2016), based on the efforts of supply chain enterprises to reduce carbon emissions, should guide enterprises to reduce emissions and give priority to subsidies rather than taxes.

According to the research of the above scholars, subsidies can better encourage enterprises to participate in the low-carbon market and improve the level of carbon emissions reduction of enterprises (Zhang *et al.,* 2016). When the government knows that enterprises apply for carbon tax incentives, it will not implement unnecessary and complicated review procedures to maximize the real social benefits. However, in carbon emissions reduction, supply chain enterprises focus on carbon tax and consumers' carbon emissions sensitivity (Guo *et al.,* 2020). Madani and Barzoki (2017) established a decision-making model in which the government acted as the leader and the green and nongreen supply chains acted as followers. The conclusion showed that the increase of subsidies led to the greening of products and the increase of profits of the government and supply chain enterprises. Government subsidies will be phased out as the market stabilizes (Zhao *et al.,* 2020).

To sum up, scholars have made a lot of fruitful studies on government subsidies and carbon emissions reduction decisions of supply chain enterprises, but they have neglected the existence of free riding behavior among supply chain enterprises, and it is easy to have speculative psychology in two cooperative or competitive enterprises. However, most of the studies on free riding behavior are in the free market, ignoring the regulatory role of the government, and there are few studies on the combination of government regulation and free riding behavior. In view of this, this article constructs a three-party carbon emissions reduction game model of the government, manufacturers and suppliers, and adopts the evolutionary game method to explore the impact of free riding behavior on the carbon emissions reduction decisions of manufacturers and supply chains under government subsidies, which has practical significance for the carbon emissions reduction decisions of manufacturers and suppliers.

## Description and Construction of Models

### Model description

This article mainly studies the two-echelon supply chain structure of manufacturers and suppliers in a low-carbon market and considers self-profit and government subsidy to decide whether to reduce carbon emissions. The manufacturers and suppliers cooperate to form a supply chain alliance in response to the government's carbon reduction policy. As the leader of the low-carbon market, the government subsidizes the enterprises that reduce emissions through the carbon incentive policy when the market mood is low, and does not adopt the policy when the market mood is high.

If the government chooses the participation strategy, it will subsidize *S* to the manufacturers and suppliers who are actively involved in carbon emissions reduction, and pay the cost of publicity and implementation *C_E_* when guiding the low-carbon market. If the government chooses the nonparticipation strategy, the manufacturers and suppliers will not actively contribute to carbon emissions reduction and will bring certain reputation loss *L* to the government. In general, the government is more concerned with the reputation. So $L>C_{E}$. No matter which strategy the government adopts, as long as either the manufacturers or the suppliers carry on technology transformation for carbon emissions reduction, the government will obtain certain national economic benefits *B*.

In the supply chain alliance, only one side of the manufacturers and suppliers is active in investment in carbon emissions reduction, and the other side can enjoy the benefits without investment in carbon emissions reduction, which constitutes the free riding effect. Based on this, the following assumptions are made:

(1) Consumers are rational and tend to choose low-carbon products.
(2) When neither the manufacturers nor the suppliers make an investment in carbon emissions reduction, the manufactured products are generic product, and the manufacturers and the suppliers have a combined profit *V_M_* and *V_S_*, of which $V_{M}>0$ and $V_{S}>0$. If government adopts participation strategy, manufacturers and suppliers suffer reputation loss *T* because they do not respond positively to the policy.
(3) When only the manufacturers actively invest in carbon emissions reduction, the manufacturers will get profits $\left(1+\alpha_{0}\right)V_{M}+S-C_{M}$, where $\alpha_{0}\left(\alpha_{0}>0\right)$ refers to the profit rate brought by the manufacturers' investment in carbon emissions reduction, and *C_M_* refers to the input cost of the manufacturers' carbon emissions reduction. In addition, the suppliers do not make investment in carbon emissions reduction but enjoy the benefits. The manufacturers' investment in carbon emissions reductions is $\pi_{S}$, $\pi_{S}>V_{S}$.
Similarly, when only the suppliers actively invest in carbon emissions reduction, the suppliers' income is $\left(1+\beta_{0}\right)V_{S}+S-C_{S}$, where $\beta_{0}\left(\beta_{0}>0\right)$ refers to the profit rate brought by the suppliers' investment in carbon emissions reduction, and *C_S_* refers to the cost of the suppliers' investment in carbon emissions reduction. Additionally, the manufacturers do not carry on the investment in carbon emissions reduction but enjoy the benefit. The suppliers' investment in carbon emissions reduction is $\pi_{M}$, and $\pi_{M}>V_{M}$.
(4) When both the manufacturers and suppliers commit to carbon emissions reduction, their returns are $\left(1+\alpha_{1}\right)V_{M}+S-C_{M}$ and $\left(1+\beta_{1}\right)V_{S}+S-C_{S}$, respectively, $\alpha_{1}$ refers to the profit rate brought to the manufacturers by both the manufacturers and suppliers' investment in carbon emissions reduction, and $\beta_{1}$ refers to the profit rate brought to the suppliers by both the manufacturers and suppliers' investment in carbon emissions reduction, where $\alpha_{1}>\alpha_{0}$ and $\beta_{1}>\beta_{0}$.
(5) Assuming that the probability of the government to adopt the participation strategy is *x*, and the probability of adopting the nonparticipation strategy is 1−x. The probability of the manufacturers' strategy of investing in carbon emissions reduction is *y*, and the probability of the manufacturers' strategy of not investing in carbon emissions reduction is 1−y. The probability that the suppliers choose the strategy of investing in carbon emissions reduction is *z*, and the probability that the suppliers choose the strategy of not investing in carbon emissions reduction is 1−z.

To be specific, we summarize the model parameters and decision variables in Table 1.

**Table 1.** Notations for Parameters and Variables

Model parameters	
*S*	Government subsidies to companies that reduce carbon emissions
*C_E_*	Government participation to cover the costs of advocacy and implementation
*L*	Reputation loss to the government when the government does not participate in the carbon emissions reduction without the enterprise
*B*	National economic benefits brought by enterprises' carbon emissions reduction to the government
*V_M_*	Manufacturers' earnings from the production of ordinary products
$\alpha_{1}$	Profitability of manufacturers when both manufacturers and suppliers reduce carbon emissions
$\alpha_{0}$	Profitability of manufacturers when only manufacturers reduce carbon emissions
*C_M_*	Cost of carbon emissions reduction for manufacturers
$\pi_{M}$	Free riding profits for manufacturers
*V_S_*	Suppliers' earnings from the production of ordinary products
$\beta_{1}$	Profitability of suppliers when both manufacturers and suppliers reduce carbon emissions
$\beta_{0}$	Profitability of suppliers when only suppliers reduce carbon emissions
*C_S_*	Cost of carbon emissions reduction for suppliers
$\pi_{S}$	Free riding profits for suppliers
*T*	Manufacturers and suppliers do not respond to calls for loss of reputation when government participates
Decision variables	
*x*	Probability of the government chooses to participate in the strategy
*y*	Probability of the manufacturers choose to reduce carbon emissions
*z*	Probability of the suppliers choose to reduce carbon emissions

### Model construction

According to the above description and hypothesis of the model, the payoff matrix of the tripartite game is shown in the following table:

## Evolutionary Game Equilibrium

Unlike the traditional game theory, which requires neither complete rationality nor complete information, the evolutionary game theory is a game theory that combines a game theory analysis with a dynamic evolution. For the participants in the social economy, the conditions of perfect rationality and perfect information cannot be realized, so the evolutionary game is realistic. The method of the evolutionary game theory is: on the basis of the dynamic equations of the government, the manufacturers and the suppliers, the Jacobian matrix is obtained by taking the partial derivatives of the unknowns *x*, *y*, and *z*, and then the local stability is analyzed according to the values and traces of the Jacobian matrix. It is determined whether the government, manufacturers, and suppliers' strategy are a stability strategy.

### Construction of expected return function

According to Tables 2 and 3, the government chooses the expected return function of participation strategy $E{\left(G\right)}_{x}$, the expected return function of nonparticipation strategy $E{\left(G\right)}_{1-x}$, and the average expected return function $\bar{E}\left(G\right)$ are:

**Table 2.** Government (Participation), Manufacturers' and Suppliers' Revenue Matrix

	Suppliers	
	Investing in carbon emissions reduction (z)	Not investing in carbon emissions reduction (*1−z*)
Manufacturers		
Investing in carbon emissions reduction (*y*)	$B-C_{E}-2S$	$B-C_{E}-S$
	$\left(1+\alpha_{1}\right)V_{M}+S-C_{M}$	$\left(1+\alpha_{0}\right)V_{M}+S-C_{M}$
	$\left(1+\beta_{1}\right)V_{S}+S-C_{S}$	$\pi_{S}$
Not investing in carbon emissions reduction (1−y)	$B-C_{E}-S$	$-C_{E}$
	$\pi_{M}$	$V_{M}-T$
	$\left(1+\beta_{0}\right)V_{S}+S-C_{S}$	$V_{S}-T$

**Table 3.** Government (Nonparticipation), Manufacturers' and Suppliers' Revenue Matrix

	Suppliers	
	Investing in carbon emissions reduction (z)	Not investing in carbon emissions reduction (*1−z*)
Manufacturers		
Investing in carbon emissions reduction (*y*)	*B*	*B*
	$\left(1+\alpha_{1}\right)V_{M}-C_{M}$	$\left(1+\alpha_{0}\right)V_{M}-C_{M}$
	$\left(1+\beta_{1}\right)V_{S}-C_{S}$	$\pi_{S}$
Not investing in carbon emissions reduction (1−y)	*B*	−L
	$\pi_{M}$	*V_M_*
	$\left(1+\beta_{0}\right)V_{S}-C_{S}$	*V_S_*


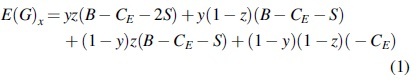



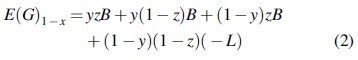


(3) $$Ē\left(G\right)=xE{\left(G\right)}_{x}+\left(1-x\right)E{\left(G\right)}_{1-x}$$

In the game, the manufacturers choose the expected revenue function of the strategy of investing in carbon emissions reduction $E{\left(M\right)}_{y}$, the expected revenue function of the strategy of not investing in carbon emissions reduction $E{\left(M\right)}_{1-y}$ and the average expected revenue function $Ē\left(M\right)$ are:

(4) $$\begin{array}{cc} E{\left(M\right)}_{y}=xz\left[\left(1+\alpha_{1}\right)V_{M}+S-C_{M}\right]+x\left(1-z\right)\\ \left[\left(1+\alpha_{0}\right)V_{M}+S-C_{M}\right]+\left(1-x\right)z\left[\left(1+\alpha_{1}\right)V_{M}-C_{M}\right]\\ & +\left(1-x\right)\left(1-z\right)\left[\left(1+\alpha_{0}\right)V_{M}-C_{M}\right] \end{array}$$

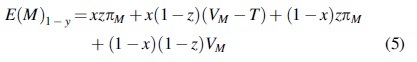


(6) $$Ē\left(M\right)=yE{\left(M\right)}_{y}+\left(1-y\right)E{\left(M\right)}_{1-y}$$

In the game, the suppliers choose the expected revenue function of the strategy of investing in carbon emissions reduction $E{\left(S\right)}_{z}$, the expected revenue function of the strategy of not investing in carbon emissions reduction $E{\left(S\right)}_{1-z}$, and the average expected revenue function $Ē\left(S\right)$ are:

(7) $$\begin{array}{cc} E{\left(S\right)}_{z}=xy\left[\left(1+\beta_{1}\right)V_{S}+S-C_{S}\right]\\ +x\left(1-y\right)\left[\left(1+\beta_{0}\right)V_{S}+S-C_{S}\right]+\\ & \left(1-x\right)y\left[\left(1+\beta_{1}\right)V_{S}-C_{S}\right]\\ +\left(1-x\right)\left(1-y\right)\left[\left(1+\beta_{0}\right)V_{S}-C_{S}\right] \end{array}$$

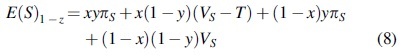


(9) $$Ē\left(S\right)=zE{\left(S\right)}_{z}+\left(1-z\right)E{\left(S\right)}_{1-z}$$

### Solution to the duplicate dynamic equation

According to the above expected revenue function, the replicate dynamic equation of the government is obtained as follows:

(10) $$\begin{array}{cc} & F\left(x\right)=\frac{dx}{dt}=x\left[E{\left(G\right)}_{x}-Ē\left(G\right)\right]\\ & =x\left(1-x\right)\left[E{\left(G\right)}_{x}-E{\left(G\right)}_{1-x}\right]\\ & =x\left(1-x\right)\left[yzL-\left(y+z\right)\left(S+L\right)-C_{E}+L\right] \end{array}$$

**FIG. 1.**
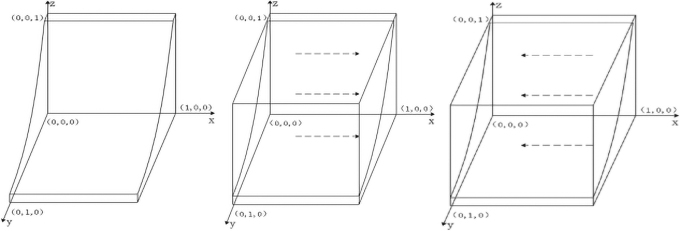
Dynamic evolution diagram of government group decision making.

By the nature of the replicated dynamic equation, (1): when $y=\frac{L-C_{E}-z\left(S+L\right)}{S+L-zL}$, $F\left(x\right)=0$ means that for any *x*, the copy dynamic equation is 0 and in a stable state; (2): when $0<y<\frac{L-C_{E}-z\left(S+L\right)}{S+L-zL}$, $F^{\prime }\left(x\right){|}_{x=0}>0$, and $F^{\prime }\left(x\right){|}_{x=1}<0$ represent x=1 as the equilibrium point, that is, the proportion of the manufacturers choosing investment in carbon emissions reduction is smaller than $\frac{L-C_{E}-z\left(S+L\right)}{S+L-zL}$, and the government will adopt the strategy of participation; (3): when $y>\frac{L-C_{E}-z\left(S+L\right)}{S+L-zL}$, $F^{\prime }\left(x\right){|}_{x=0}<0$, and $F^{\prime }\left(x\right){|}_{x=1}>0$ represent x=0 as the equilibrium point, that is, the proportion of manufacturers choosing investment in carbon emissions reduction is greater compared with $\frac{L-C_{E}-z\left(S+L\right)}{S+L-zL}$, and the government will adopt the strategy of nonparticipation. The dynamic evolution diagram of government group decision making is shown in Fig. 1.

The replicate dynamic equation of the manufacturers is obtained as follows:


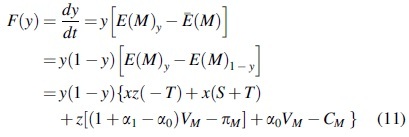


**FIG. 2.**
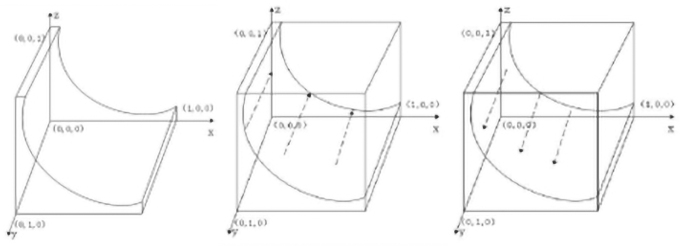
Dynamic evolution diagram of manufacturer group decision making.

By the nature of the replicated dynamic equation, (1): when $z=\frac{C_{M}-x\left(S+T\right)-\alpha_{0}V_{M}}{\left(1+\alpha_{1}-\alpha_{0}\right)V_{M}-\pi_{M}-xT}$,

$F\left(y\right)=0$ means that for any *y*, the replicated dynamic equation is 0 and in a stable state; (2): when $0<z<\frac{C_{M}-x\left(S+T\right)-\alpha_{0}V_{M}}{\left(1+\alpha_{1}-\alpha_{0}\right)V_{M}-\pi_{M}-xT}$, $F^{\prime }\left(y\right){|}_{y=0}<0$ and $F^{\prime }\left(y\right){|}_{y=1}>0$ represent y=0 as the equilibrium point, that is, when the proportion of suppliers choosing investment in carbon emissions reduction is less than $\frac{C_{M}-x\left(S+T\right)-\alpha_{0}V_{M}}{\left(1+\alpha_{1}-\alpha_{0}\right)V_{M}-\pi_{M}-xT}$, the manufacturers will adopt the strategy of no investment in carbon emissions reduction; (3): when $z>\frac{C_{M}-x\left(S+T\right)-\alpha_{0}V_{M}}{\left(1+\alpha_{1}-\alpha_{0}\right)V_{M}-\pi_{M}-xT}$, $F^{\prime }\left(y\right){|}_{y=0}>0$, and $F^{\prime }\left(y\right){|}_{y=1}<0$ represent y=1 as the equilibrium point, that is, the proportion of the suppliers choosing investment in carbon emissions reduction is greater compared with $\frac{C_{M}-x\left(S+T\right)-\alpha_{0}V_{M}}{\left(1+\alpha_{1}-\alpha_{0}\right)V_{M}-\pi_{M}-xT}$, and the manufacturers will adopt the strategy of investment in carbon emissions reduction. The dynamic evolution diagram of manufacturers' group decision making is shown in Fig. 2.

The replicate dynamic equation of the suppliers is obtained as follows:


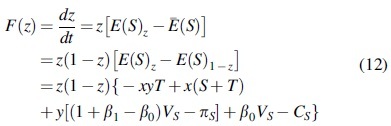


**FIG. 3.**
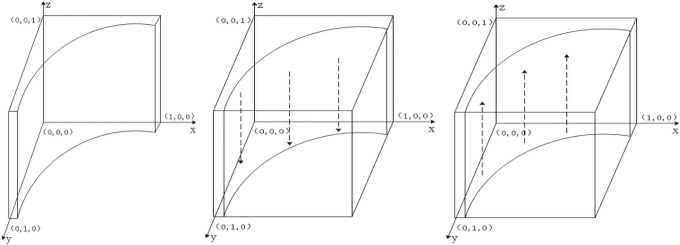
Dynamic evolution diagram of supplier group decision making.

By the nature of the replicated dynamic equation, (1): when $y=\frac{C_{S}-\beta_{0}V_{S}-x\left(S+T\right)}{\left(1+\beta_{1}-\beta_{0}\right)V_{S}-\pi_{S}-xT}$, $F\left(z\right)=0$ means that for any *y*, the replicated dynamic equation is 0 and in a stable state; (2): when $0<y<\frac{C_{S}-\beta_{0}V_{S}-x\left(S+T\right)}{\left(1+\beta_{1}-\beta_{0}\right)V_{S}-\pi_{S}-xT}$, $F^{\prime }\left(z\right){|}_{z=0}<0$, and $F^{\prime }\left(z\right){|}_{z=1}>0$ represent z=0 as the equilibrium point, that is, the proportion of manufacturers choosing investment in carbon emissions reduction is smaller compared with $\frac{C_{S}-\beta_{0}V_{S}-x\left(S+T\right)}{\left(1+\beta_{1}-\beta_{0}\right)V_{S}-\pi_{S}-xT}$, and suppliers choose the strategy of no investment in carbon emissions reduction; (3): when $y>\frac{C_{S}-\beta_{0}V_{S}-x\left(S+T\right)}{\left(1+\beta_{1}-\beta_{0}\right)V_{S}-\pi_{S}-xT}$, $F^{\prime }\left(z\right){|}_{z=0}<0$ and $F^{\prime }\left(z\right){|}_{z=1}>0$ represent z=0 as the equilibrium point, that is, the proportion of manufacturers choosing investment in carbon emissions reduction is greater compared with $\frac{C_{S}-\beta_{0}V_{S}-x\left(S+T\right)}{\left(1+\beta_{1}-\beta_{0}\right)V_{S}-\pi_{S}-xT}$, and suppliers choose the strategy of investment in carbon emissions reduction. The dynamic evolution diagram of suppliers' group decision making is shown in Fig. 3.

### Analysis of evolutionary game equilibrium

According to the local stability analysis of the above differential Equations (10–12), the Jacobian matrix of the system is:

$$J=\left[\begin{array}{ccc} a_{11} & a_{12} & a_{13}\\ a_{21} & a_{22} & a_{23}\\ a_{31} & a_{32} & a_{33} \end{array}\right]$$

$$a_{11}=\left(1-2x\right)\left[yzL-\left(y+z\right)\left(S+L\right)-C_{E}+L\right]$$

$$a_{12}=x\left(1-x\right)\left(zL-S-L\right)$$

$$a_{13}=x\left(1-x\right)\left(yL-S-L\right)$$

$$a_{21}=y\left(1-y\right)\left\{-zT+S+T\right\}$$

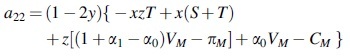


$$a_{23}=y\left(1-y\right)\left[-xT+\left(1+\alpha_{1}-\alpha_{0}\right)V_{M}-\pi_{M}\right]$$

$$a_{31}=z\left(1-z\right)\left(-yT+S+T\right)$$

$$a_{32}=z\left(1-z\right)\left\{-xT+\left(\beta_{1}-\beta_{0}\right)V_{S}-\pi_{S}+V_{S}\right\}$$

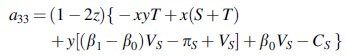


Through a Jacobian matrix analysis, it can be seen that the equilibrium point of the evolutionary game between the government, manufacturers, and suppliers are: $e_{1}\left(0,0,0\right)$, $e_{2}\left(0,1,0\right)$, $e_{3}\left(0,0,1\right)$, $e_{4}\left(0,1,1\right)$, $e_{5}\left(1,0,0\right)$, $e_{6}\left(1,0,1\right)$, $e_{7}\left(1,1,0\right)$, and $e_{8}\left(1,1,1\right)$. According to the evolutionary game theory, when all eigenvalues of Jacobian matrix are not positive, the equilibrium point is a stable point.

Let's first analyze the Jacobian matrix in the case of $e_{1}\left(0,0,0\right)$.

$$J_{1}=\left[\begin{array}{ccc} L-C_{E} & 0 & 0\\ 0 & \alpha_{0}V_{M}-C_{M} & 0\\ 0 & 0 & \beta_{0}V_{S}-C_{S} \end{array}\right]$$

As can be seen above, the eigenvalue of *J*_1_ is: $\lambda_{1}=L-C_{E}$, $\lambda_{2}=\alpha_{0}V_{M}-C_{M}$, and $\lambda_{3}=\beta_{0}V_{S}-C_{S}$. Similarly, eigenvalues of the remaining equilibrium points can be obtained, as shown in Table 4.

**Table 4.** Eigenvalues of Jacobian Matrix

Equilibrium	Eigenvalue $\lambda_{1}$	Eigenvalue $\lambda_{2}$	Eigenvalue $\lambda_{3}$
$e_{1}\left(0,0,0\right)$	$L-C_{E}$	$\alpha_{0}V_{M}-C_{M}$	$\beta_{0}V_{S}-C_{S}$
$e_{2}\left(0,1,0\right)$	$-S-C_{E}$	$C_{M}-\alpha_{0}V_{M}$	$\left(1+\beta_{1}\right)V_{S}-\pi_{S}-C_{S}$
$e_{3}\left(0,0,1\right)$	$-S-C_{E}$	$\left(1+\alpha_{1}\right)V_{M}-C_{M}-\pi_{M}$	$C_{S}-\beta_{0}V_{S}$
$e_{4}\left(0,1,1\right)$	$-2S-C_{E}$	$-\left(1+\alpha_{1}\right)V_{M}+C_{M}+\pi_{M}$	$-\left(1+\beta_{1}\right)V_{S}+\pi_{S}+C_{S}$
$e_{5}\left(1,0,0\right)$	$C_{E}-L$	$S+T+\alpha_{0}V_{M}-C_{M}$	$S+T+\beta_{0}V_{S}-C_{S}$
$e_{6}\left(1,0,1\right)$	$S+C_{E}$	$\left(1+\alpha_{1}\right)V_{M}+S-C_{M}-\pi_{M}$	$C_{S}-S-T-\beta_{0}V_{S}$
$e_{7}\left(1,1,0\right)$	$S+C_{E}$	$C_{M}-S-T-\alpha_{0}V_{M}$	$\left(1+\beta_{1}\right)V_{S}+S-\pi_{S}-C_{S}$
$e_{8}\left(1,1,1\right)$	$2S+C_{E}$	$\pi_{M}+C_{M}-\left(1+\alpha_{1}\right)V_{M}-S$	$C_{S}+\pi_{S}-\left(1+\beta_{1}\right)V_{S}-S$

To facilitate the analysis of the positive and negative signs of eigenvalues at different equilibrium points without loss of generality, the evolutionary game is discussed in the following four cases:

Case 1: when $\alpha_{0}>\frac{C_{M}}{V_{M}}$ and $\beta_{0}<\beta_{1}<\frac{\pi_{S}+C_{S}-V_{S}}{V_{S}}$, only to its suppliers for carbon emissions reduction in yield is too small, it cannot satisfy the suppliers' expected return, and only to the manufacturers for carbon emissions reduction in yield to meet manufacturers' expected profits, so manufacturers choose commitments to carbon emissions reduction, suppliers choose no commitments to carbon emissions reduction, but enjoy the free riding effect. At this point, $\left(0,1,0\right)$ is the equilibrium point of the evolutionary game.

Case 2: when $\alpha_{0}<\alpha_{1}<\frac{C_{M}+\pi_{M}-V_{M}}{V_{M}}$ and $\beta_{0}>\frac{C_{S}}{V_{S}}$, only to the manufacturers for carbon emissions reduction in yield is too small, it cannot satisfy the manufacturers' expected revenue, and only to its suppliers for carbon emissions reduction in yield to meet suppliers' expected profits, so suppliers choose commitments to carbon emissions reduction, and manufacturers choose no commitments to carbon emissions reduction commitments, but enjoy the free riding effect. At this point, $\left(0,0,1\right)$ is the equilibrium point of the evolutionary game.

Case 3: when $\alpha_{1}>\frac{C_{M}+\pi_{M}-V_{M}}{V_{M}}$ and $\beta_{1}>\frac{\pi_{S}+C_{S}-V_{S}}{V_{S}}$, the profitability brought by both the manufacturers and the suppliers' investment in carbon emissions reduction is large enough, therefore, both the manufacturers and the suppliers choose the strategy of investment in carbon emissions reduction. At this point, $\left(0,1,1\right)$ is the equilibrium point of the evolutionary game.

Case 4: when $\alpha_{0}<\frac{C_{M}-S-T}{V_{M}}$ and $\beta_{0}<\frac{C_{S}-S-T}{V_{S}}$, only the manufacturers or suppliers' investment in carbon emissions reduction brings too small an earning rate to attract the manufacturers or suppliers' investment in carbon emissions reduction. At this point, $\left(1,0,0\right)$ is the equilibrium point of the evolutionary game. The government chooses the strategy of participation, whereas manufacturers and suppliers choose the strategy of no investment in carbon emissions reduction. According to the research by other scholars, with the gradual stability of the market, the government will eventually withdraw gradually. Therefore, Case 4 will not be discussed below.

## Numerical Simulation

To better show the evolution path of the system, a data simulation analysis is carried out on the above model. There is a denim clothing manufacturer, Company F in the Pearl River Delta, which forms a supply chain with upstream fabric accessories' suppliers. According to the research on investment in carbon emissions reduction of Company F's supply chain products, the rate of return of Company F's investment in carbon emissions reduction alone is $\alpha_{0}\in \left[0.235,0.263\right]$. The earning rate of investment in carbon emissions reduction of both sides is $\alpha_{1}\in \left[0.361,0.385\right]$, which meets $\alpha_{0}<\alpha_{1}$. The rate of return brought by the suppliers' investment in carbon emissions reduction alone is $\beta_{0}\in \left[0.228,0.243\right]$, and the rate of return brought by both parties' investment in carbon emissions reduction is $\beta_{1}\in \left[0.452,0.464\right]$, which meets $\beta_{0}<\beta_{1}$. The cost of investment in carbon emissions reduction by Company F is $C_{M}\in \left[1350,1450\right]$, and the cost investment in carbon emissions reduction by its suppliers is $C_{S}\in \left[800,900\right]$. When Company F and its suppliers do not make an investment in carbon emissions reduction, the income of Company F is $V_{M}\in \left[5700,6200\right]$, and the benefit of free riding is $\pi_{M}\in \left[7114,7190\right]$, the income of its suppliers is $V_{S}\in \left[3500,4100\right]$, and the benefit of free riding is $\pi_{S}\in \left[5154,5292\right]$.

When the government chooses the strategy of participation, the subsidy *S* is 280 for manufacturers and suppliers who actively invest in carbon emissions reduction. When guiding the low-carbon market, the cost of publicity and implementation *C_E_* is 200. If manufacturers and suppliers do not actively respond to the policy, the reputation loss *T* is 300. When the government chooses the strategy of nonparticipation, manufacturers and suppliers do not invest in carbon emissions reduction, which will bring the government a reputation loss of *L*, 300.

### Analysis of one subject's investment in carbon emissions reduction

#### Analysis of free riding motivation of manufacturers and suppliers

It is clear that when the rate of return of c investment in carbon emissions reduction by manufacturers and suppliers meets $\frac{C_{M}}{V_{M}}<\alpha_{0}<\alpha_{1}<\frac{C_{M}+\pi_{M}-V_{M}}{V_{M}}$, $\frac{C_{S}}{V_{S}}<\beta_{0}<\beta_{1}<\frac{C_{S}+\pi_{S}-V_{S}}{V_{S}}$. Manufacturers and suppliers have free riding motives. The result of evolution is $\left(x,1,0\right)$ or $\left(x,0,1\right)$(x indicating an uncertain government strategy). The final convergence of the system is affected by the input cost, the income from the investment in carbon emissions reduction, and the income from the free riding behavior.

**FIG. 4.**
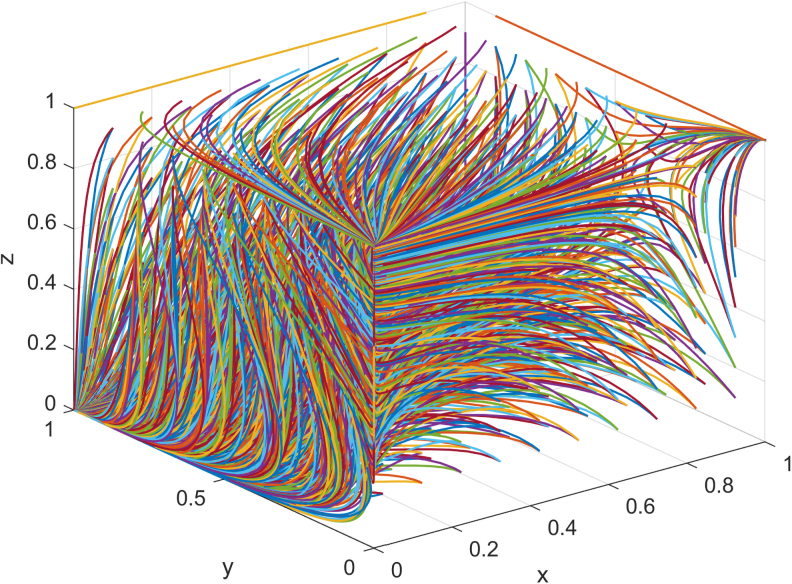
Evolutionary path of three-party game under one subject.

Combined with the actual operation of Company F's supply chain of jeans manufacturing, the given values are as follows: the earning rate of Company F's investment in carbon emissions reduction $\alpha_{0}$ is 0.26, of both sides $\alpha_{1}$ is 0.38, of its suppliers $\beta_{0}$ is 0.23, and that of both sides $\beta_{1}$ is 0.46. The cost of Company F's investment in carbon emissions reduction *C_M_* is 1,350, and the cost of suppliers' investment in carbon emissions reduction *C_S_* is 800. When Company F and suppliers do not make an investment in carbon emissions reduction, Company F's income *V_M_* is 5,700, and suppliers' income *V_S_* is 3,500. Taking free riding behavior brings $\pi_{M}$ 7,114 to manufacturers and $\pi_{S}$ 5,154 to suppliers. The evolution result is shown in Fig. 4.

**FIG. 5.**
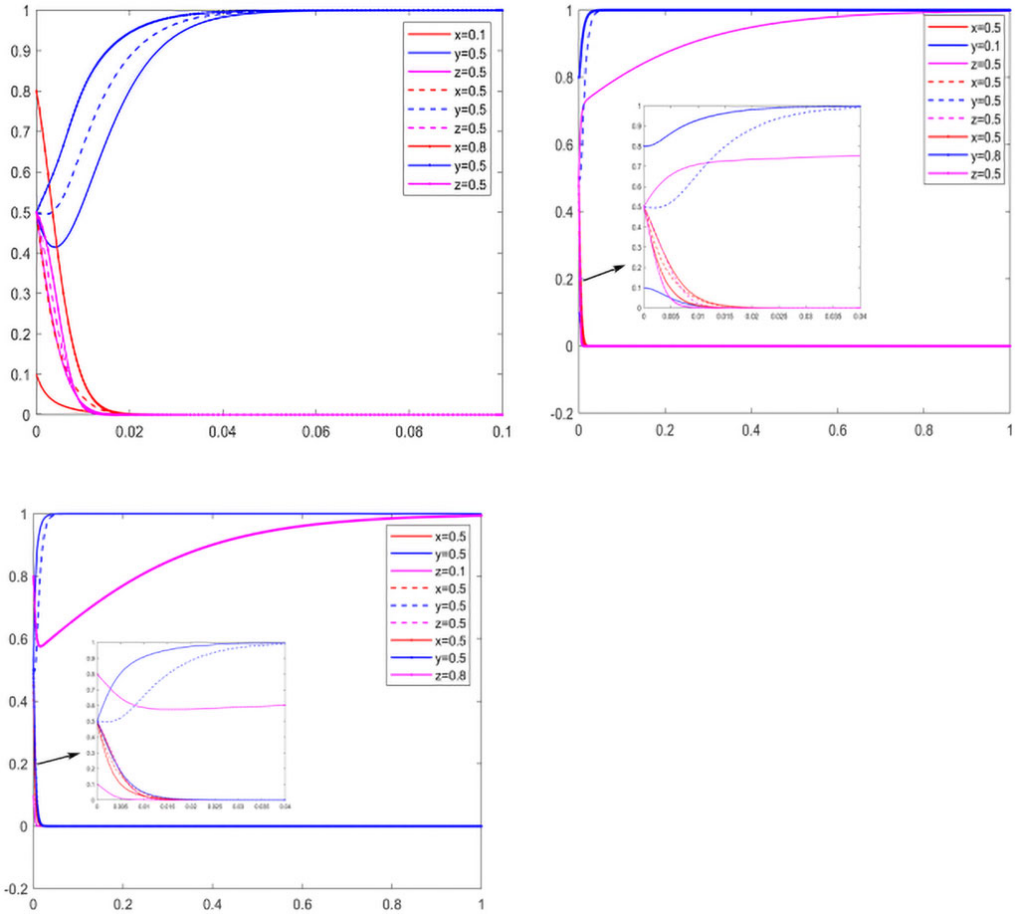
Influence of initial intention change under one subject.

As can be seen from Fig. 4, the probability of the government adopting the strategy of nonparticipation is getting higher and higher. To obtain higher benefits, both the manufacturers and suppliers hope to reduce the cost of investment in carbon emissions reduction through free riding behavior, so as to obtain the benefits brought by carbon emissions reduction, and finally evolve into $\left(0,1,0\right)$ or $\left(0,0,1\right)$. In the long run, without government supervision, it is inevitable that the manufacturers and suppliers will choose to invest in carbon emissions reduction, which is the goal of China's green development. By assigning the initial values *x*, *y*, *z*, we simulate the impact of one party's willingness to reduce carbon emissions on the other two parties who maintain a neutral attitude, and get Fig. 5.

As can be seen from Fig. 5a, when the initial willingness of the government to encourage enterprises to invest in carbon emissions reduction is high, Company F immediately responds to the policy of investment in carbon emissions reduction. With the maturity of the low-carbon market, the government gradually withdraws from the market of carbon emissions reduction. Facing the positive carbon emissions reduction of F company, the suppliers choose not to carry on the carbon emissions reduction, reduce the cost of carbon emissions reduction while maintaining profit growth. When the initial willingness of the government to encourage enterprises to invest in carbon emissions reduction is low, Company F changes from being unwilling to invest in carbon emissions reduction to actively investing in carbon emissions reduction, and the convergence rate of *x* and *z* gradually decreases.

It can be seen from Fig. 5b that when Company F has a high initial willingness to invest in carbon emissions reduction, with the increase in *y*, the convergence rate of *x* and *z* gradually slows down, the willingness of the government and suppliers to invest in carbon emissions reduction is not high, and the low-carbon market is dominated by manufacturers. When Company F's initial willingness to invest in carbon emissions reduction is low, to obtain the low-carbon market, the suppliers become the leader for investment in carbon emissions reduction. With the decrease in *y*, the convergence rate of *x* increases and the convergence rate of *z* decreases.

It can be seen from Fig. 5c that when the initial willingness of suppliers to invest in carbon emissions reduction is high, Company F is unwilling to invest in carbon emissions reduction, and the government also adopts the participation strategy. Suppliers' willingness gradually changes from high to neutral attitude. As the convergence rate of willingness of Company F and the government to invest in carbon emissions reduction decreases, the willingness of suppliers to reduce carbon emissions increases. When the initial willingness of suppliers to invest in carbon emissions reduction is low, Company F is more willing to invest in carbon emissions reduction. With the decrease in the convergence rate *z*, the convergence rate of *x* is changing from a higher one to a lower one.

#### Sensitivity analysis for earning rate

Manufacturers and suppliers consider the benefits of reducing carbon emissions and the level of government subsidy when making decisions. It is assumed that the government, manufacturers, and suppliers initially intend to remain neutral, analyzing the sensitivity of $\alpha_{0}$, $\alpha_{1}$, $\beta_{0}$, and $\beta_{1}$.

**FIG. 6.**
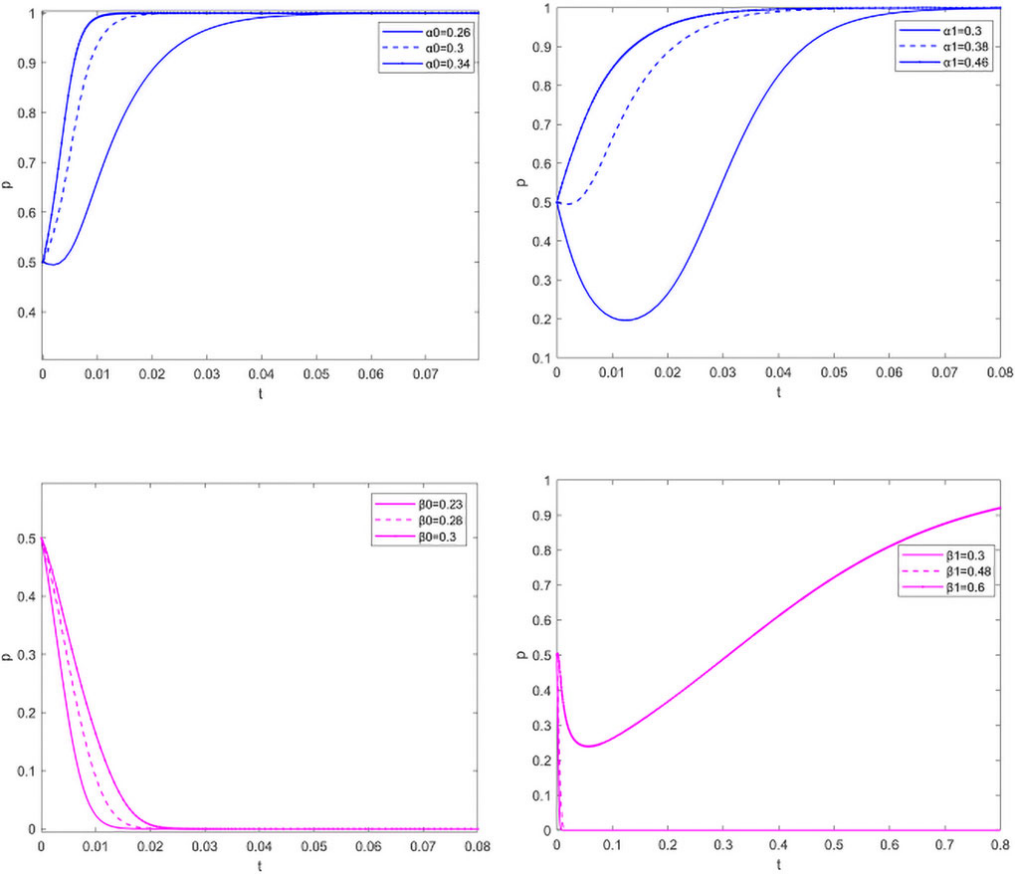
Sensitivity analysis for earning rate.

As can be seen from Fig. 6a, when it comes to reducing carbon emissions, manufacturers, as the dominant player in the market, will not care about as long as the free riding behavior of suppliers is not serious. The higher the earning rate of carbon emissions reduction, the stronger the manufacturers' carbon emissions reduction will be. However, the higher the earning rate obtained by the suppliers from carbon emissions reduction alone, the stronger its reluctance to carry out carbon emissions reduction will be. As the earning rate of carbon emissions reduction alone is close to the carbon emissions reduction of both parties, suppliers as market followers, are unwilling to bear the cost of carbon emissions reduction alone, and think that the free riding behavior of manufacturers is too serious (Fig. 6c). When the earning rate from carbon emissions reduction increases for both parties, the higher the earning rate, and the stronger the manufacturers will be in carbon emissions reduction (Fig. 6b). For suppliers, they are only willing to reduce carbon emissions when the earning rate exceeds a certain threshold (Fig. 6d). As can be seen from Fig. 6, when making decisions, manufacturers value the earning rate brought by carbon emissions reduction, while suppliers choose not to carry out carbon emissions reduction in most cases. Only when carbon emissions reduction can bring considerable earning rate, will they be willing to work with manufacturers to reduce carbon emissions.

#### Sensitivity analysis for free riding behavior parameters

To further understand the free riding behavior, a sensitivity analysis was carried out on the parameters of $\pi_{S}$ and $\pi_{M}$. Assuming that the initial intentions of the government, manufacturers, and suppliers are neutral, the convergence rate will change with the change in free riding behavior parameters.

**FIG. 7.**
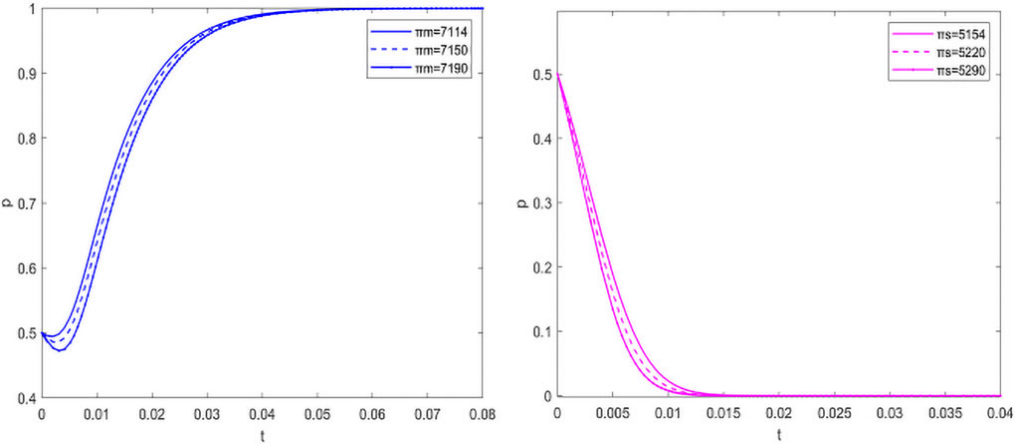
Sensitivity analysis for free riding earnings.

With the increase in the convergence rate $\pi_{M}$, the convergence rate *y* slows down. When free riding gets low benefit, the willingness of manufacturers to invest in carbon emissions reduction increases. As manufacturers, they are willing to make more profits for low-carbon emissions reduction market. When suppliers get higher free riding income, they often choose not to invest in carbon emissions reduction. With the increase in $\pi_{S}$, the convergence rate of *z* increases. As the weak side of the market, suppliers have a speculative mentality in carbon emissions reduction and are unwilling to pay the cost and enjoy the benefits of the low-carbon market (Fig. 7).

### Analysis of two subjects' investment in carbon emissions reduction

**FIG. 8.**
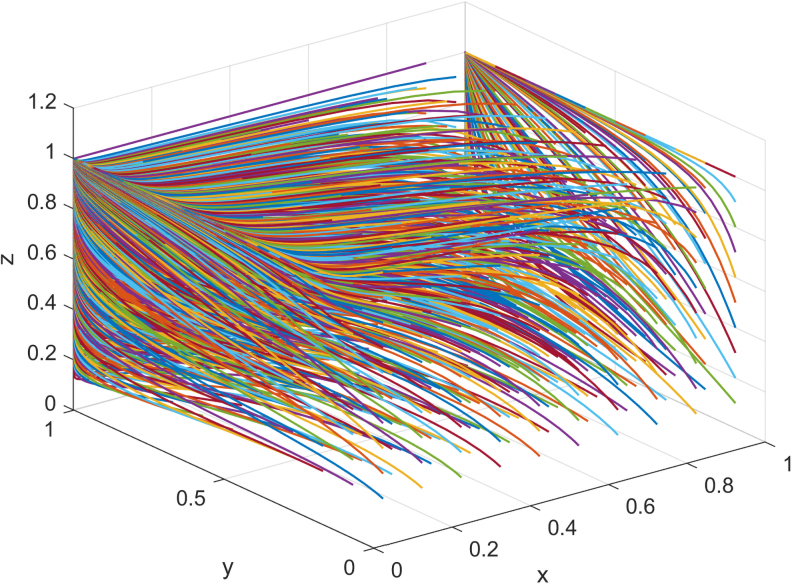
Evolutionary path of three-party game under two subjects.

According to the above research, when $\alpha_{1}>\frac{C_{M}+\pi_{M}-V_{M}}{V_{M}}$, $\beta_{1}>\frac{\pi_{S}+C_{S}-V_{S}}{V_{S}}$, both manufacturers and suppliers invest in carbon emissions reduction. Combined with the actual operation of Company F's jeans manufacturing supply chain, the given values are as follows: the earning rate of Company F's investment in carbon emissions reduction $\alpha_{0}$ is 0.26, of both sides $\alpha_{1}$ is 0.38, of its suppliers $\beta_{0}$ is 0.23, and that of both sides $\beta_{1}$ is 0.46. The cost of Company F's investment in carbon emissions reduction *C_M_* is 1,350, and the cost of suppliers' investment in carbon emissions reduction *C_S_* is 800. When Company F and suppliers do not make investment in carbon emissions reduction, Company F's income *V_M_* is 6,200, and suppliers' income *V_S_* is 4,100. Taking free riding behavior brings $\pi_{M}$ of 7,114 to the manufacturers and $\pi_{S}$ of 5,154 to the suppliers. The evolution result is shown in Fig. 8.

**FIG. 9.**
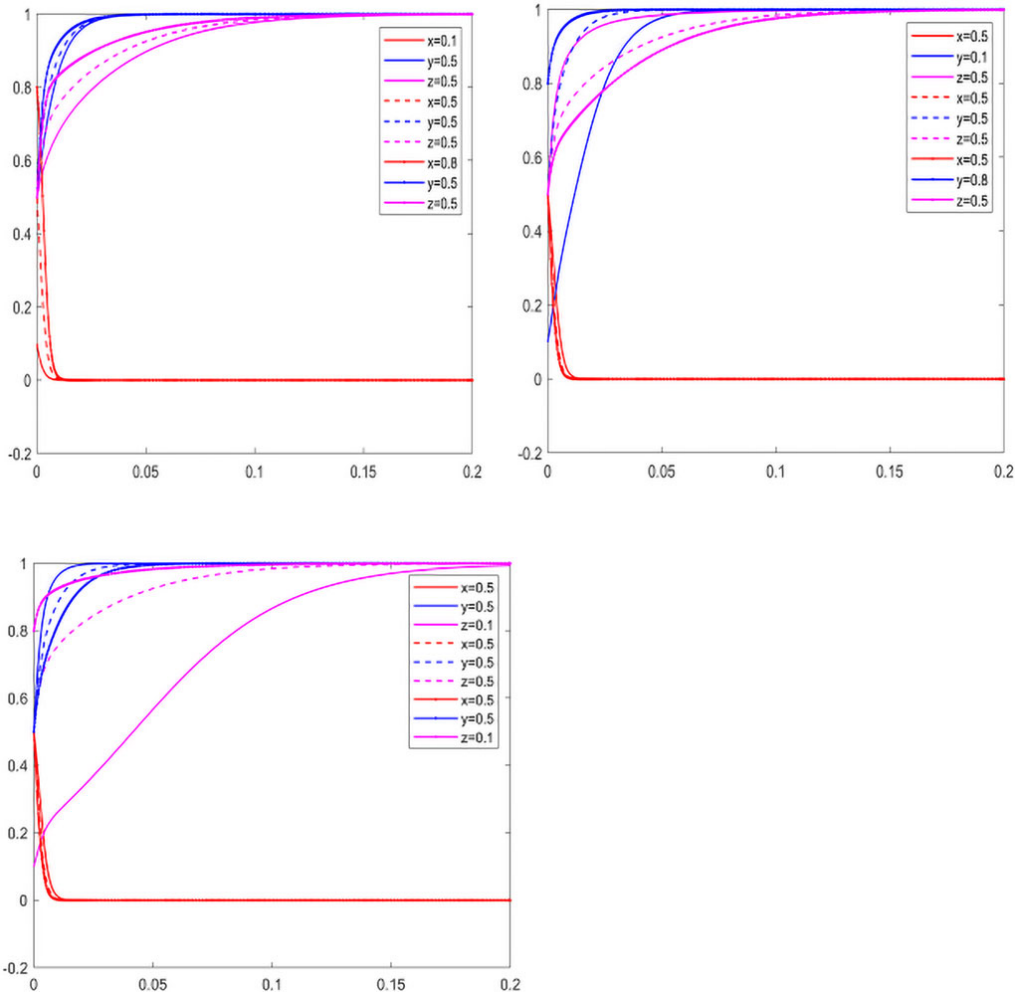
Influence of initial intention change under two subjects.

As can be seen from Fig. 8, when manufacturers and suppliers obtain more benefits through investment in carbon emissions reduction, the result of the three-party game eventually evolves into the strategy of nonparticipation by the government, whereas manufacturers and suppliers choose the strategy of investment in carbon emissions reduction. As carbon emissions reduction brings higher profits, manufacturers and suppliers do not care about each other's free riding behavior, thus the final evolution is $\left(0,1,1\right)$. By assigning initial values of *x*, *y*, and *z*, the effects of one party's willingness to reduce carbon emissions on the other two parties with a neutral attitude are simulated and shown in Fig. 9.

As can be seen from Fig. 9a, regardless of the government's willingness to encourage enterprises to invest in carbon emissions reduction, manufacturers and suppliers are willing to invest in carbon emissions reduction in the face of benefits from high carbon emissions reduction. However, the convergence rate of the willingness to invest in carbon emissions reduction slows down with the decrease in the government's willingness not to participate in the low-carbon market. As *x* decreases, the convergence rate of *y* and *z* slows down.

As can be seen from Fig. 9b, when the manufacturers have a high initial willingness to invest in carbon emissions reduction, although the suppliers can gain benefits through free riding behavior, for the sake of corporate reputation and low-carbon market share, the suppliers choose to invest in carbon emissions reduction, and their willingness to invest in carbon emissions reduction decreases. When manufacturers have a low initial willingness to invest in carbon emissions reduction, as the weak party in the market, attracted by the huge profits in the low-carbon market, they will take the initiative to undertake the task of carbon emissions reduction, regardless of whether manufacturers take the free riding behavior.

As can be seen from Fig. 9c, when the suppliers have a high initial willingness to invest in carbon emissions reduction, the manufacturers, as the market leader, are unwilling to give up the benefits of the low-carbon market and will choose to invest in carbon emissions reduction. When the suppliers' willingness to make an investment in carbon emissions reduction is low, the manufacturers will take the lead in making an investment in carbon emissions reduction, and the convergence rate is higher compared with the manufacturers when the suppliers' initial willingness is high.

## Conclusions

This article constructs a three-party carbon emissions reduction game model among the government, manufacturers, and suppliers to study the effects of government subsidies and free riding on the decision making of manufacturers and suppliers, and the evolutionary stability game strategy and the parameter sensitivity of free riding behavior are analyzed. The analysis shows that: (1) government subsidies have an incentive effect on carbon emissions reduction of supply chain enterprises. After the market stabilizes, even if the government subsidies are gradually withdrawn, the carbon emissions reduction of supply chain enterprises still converges to Pareto optimal equilibrium. (2) When the profit from carbon emissions reduction is low, for the manufacturers, as long as the free riding behavior of the suppliers is not serious, the manufacturers will be willing to carry out carbon emissions reduction. (3) When the profit of carbon emissions reduction is high, both manufacturers and suppliers are willing to carry out carbon emissions reduction regardless of whether the other party will take the free riding behavior. Although the free riding behavior will not affect the manufacturers and suppliers to carry out carbon emissions reduction, its convergence rate slows down.

For supply chain companies, government subsidies have increased profits and carbon emissions. When the government subsidizes, the enterprises carry on the carbon emissions reduction to be able to promote own development, but to be in the market, the weak enterprise is more likely to have the speculation psychology, the excess profit does not have to pay the extra cost for these weak enterprise to be full of enticement, so weak companies generally choose free riding, do not reduce carbon emissions, unless carbon emissions reduction net profit is far greater than the free riding profit. Market-oriented enterprises are more sensitive to market demand, can respond quickly to market demand, and with the rise of the concept of low-carbon environmental protection, leading enterprises are more willing to reduce carbon emissions, responding to market calls.

In this article, we study the degree of free riding effects of manufacturers and suppliers of carbon emissions reduction decision making, but only consider the government through subsidy means to carry on the market regulation, overlooked the other ways to participate in the government, such as the punishment mechanism, carbon trading policy and carbon emissions limit, in a follow-up study, tries to study the government more means of market regulation for manufacturers and suppliers of carbon emissions reduction decision-making influence.
